# A GBS-based genetic linkage map and quantitative trait loci (QTL) associated with resistance to *Xanthomonas campestris* pv. *campestris* race 1 identified in *Brassica oleracea*

**DOI:** 10.3389/fpls.2023.1205681

**Published:** 2023-06-13

**Authors:** Lu Lu, Su Ryun Choi, Yong Pyo Lim, Si-Yong Kang, So Young Yi

**Affiliations:** ^1^ Institute of Agricultural Science, Chungnam National University, Daejeon, Republic of Korea; ^2^ Molecular Genetics and Genomics Laboratory, Department of Horticulture, Chungnam National University, Daejeon, Republic of Korea; ^3^ Department of Horticulture, College of Industrial Sciences, Kongju National University, Yesan, Republic of Korea; ^4^ Research Center of Crop Breeding for Omics and Artificial Intelligence, Kongju National University, Yesan, Republic of Korea

**Keywords:** *Brassica oleracea*, black rot, quantitative trait loci, genotyping by sequencing, linkage map, *Xanthomonas campestris* pv. *campestris*

## Abstract

The production of *Brassica oleracea*, an important vegetable crop, is severely affected by black rot disease caused by the bacterial pathogen *Xanthomonas campestris* pv. *campestris*. Resistance to race 1, the most virulent and widespread race in *B. oleracea*, is under quantitative control; therefore, identifying the genes and genetic markers associated with resistance is crucial for developing resistant cultivars. Quantitative trait locus (QTL) analysis of resistance in the F_2_ population developed by crossing the resistant parent BR155 with the susceptible parent SC31 was performed. Sequence GBS approach was used to develop a genetic linkage map. The map contained 7,940 single nucleotide polymorphism markers consisting of nine linkage groups spanning 675.64 cM with an average marker distance of 0.66 cM. The F_2:3_ population (N = 126) was evaluated for resistance to black rot disease in summer (2020), fall (2020), and spring (2021). QTL analysis, using a genetic map and phenotyping data, identified seven QTLs with LOD values between 2.10 and 4.27. The major QTL, *qCaBR1*, was an area of overlap between the two QTLs identified in the 2^nd^ and 3^rd^ trials located at C06. Among the genes located in the major QTL interval, 96 genes had annotation results, and eight were found to respond to biotic stimuli. We compared the expression patterns of eight candidate genes in susceptible (SC31) and resistant (BR155) lines using qRT-PCR and observed their early and transient increases or suppression in response to *Xanthomonas campestris* pv. *campestris* inoculation. These results support the involvement of the eight candidate genes in black rot resistance. The findings of this study will contribute towards marker-assisted selection, additionally the functional analysis of candidate genes may elucidate the molecular mechanisms underlying black rot resistance in *B. oleracea*.

## 1 Introduction

*Brassica oleracea* is a plant species that includes several popular vegetables such as broccoli, cauliflower, kale, Brussels sprouts, and cabbage, which are grown and consumed worldwide. Black rot is one of the most prevalent diseases among these crops, caused by the bacterial pathogen *Xanthomonas campestris* pv. *campestris* (*Xcc*). *Xcc* affects *B. oleracea* crops, causing significant economic losses and reducing production performance and quality (Dhar and Singh, 2014). The disease can be transmitted through infected seeds, infested soil, crop residues, and various environmental and mechanical means (Vicente and Holub, 2013). *Xcc* can infect plants at any stage of development and enter through hydathodes, wounds, and other entry points. Symptoms include V-shaped yellow lesions progressing from the leaf margins to the middle vein, darkening of the veins and vascular tissue, premature leaf fall, and stunted growth (Agrios and Dawson, 2004). *Xcc* has 11 physiological races (Vicente et al., 2001; Cruz et al., 2017), with races 1 (R1) and 4 (R4) being the most aggressive and predominant worldwide (Lema et al., 2012; Vicente and Holub, 2013). Control management for black rot is limited but includes copper application, crop rotation, crop debris, cruciferous weed removal, seed treatment, and cultivation of resistant cultivars (Vicente and Holub, 2013). Among all, growing *Xcc*-resistant cultivars can help achieve sustainable and effective disease control. However, according to previous reports, the *R* genes that confer resistance to R1 are present in the B genomes of *Brassica carinata* (BBCC), *Brassica juncea* (AABB), and *Brassica nigra* (BB) (Vicente et al., 2001; Taylor et al., 2002). Currently, no major genes have been identified to be responsible for resistance to black rot in cabbage cultivars. The black rot resistance of most *B. oleracea* lines is considered to be under quantitative control (Taylor et al., 2002; Lema et al., 2012).

The identification of Quantitative Trait Loci (QTL) associated with black rot resistance in cabbage has been a subject of research for several years. Since *Xcc* R1 and R4 are considered the most virulent and widespread races in *B. oleracea*, several studies have been conducted to identify the *R*-genes/QTLs and markers linked to black rot (*Xcc* R1 and R4) resistance in *B. oleracea*. Sharma et al. identified the black rot resistance locus Xca1bc on LG B-7 in Indian mustard (*Brassica carinata*). They also reported that a single dominant gene controls black rot resistance in *B. carinata* (Sharma et al., 2016). Two sequence characterized amplified region (SCAR) markers, ScOPO-04833 and ScPKPS-11635, were identified in close linkage with the black rot resistance locus (*Xca1Bo*) in cauliflower and showed 100% accuracy in differentiating the resistant and susceptible plants of cauliflower breeding lines (Kalia et al., 2017). The identification of QTLs related to resistance to R1 of *Xcc* in cabbage has been a subject of research for several years (Camargo et al., 1995; Doullah et al., 2011; Kifuji et al., 2012; Tonu et al., 2013; Lee et al., 2015; Afrin et al., 2018; Iglesias-Bernabe et al., 2019). QTLs controlling the resistance to *Xcc* R1 have been mapped, and two significant QTLs have been identified in LG2 and LG9 in *B. oleracea* (Camargo et al., 1995; Doullah et al., 2011; Tonu et al., 2013). Kifuji et al. reported a QTL for black rot resistance located on LG C02, which comprises the major QTL in cabbage (Kifuji et al., 2012). Furthermore, researchers have mapped the *Xcc* R1 resistance locus, *Xca1bo*, on chromosome 3 in Indian cauliflowers using bulk segregant analysis, while several random amplified polymorphic DNA (RAPD) markers have been linked to *Xcc* R1 resistance locus (Saha et al., 2014a; Saha et al., 2014b). Lee et al. reported a genetic linkage map where they improved the resolution of a previously developed genetic map, and QTL analysis identified one major (*BRQTL-C1_2*) and three minor QTLs (*BRQTL-C1_1*, *BRQTL-C3*, and *BRQTL-C6*) (Lee et al., 2015). Iglesias-Bernabé et al. measured five traits related to the initial stages of invasion, success of infection, and spread of the pathogen in a BolTBDH mapping population and identified four single-trait QTLs that confirmed the quantitative nature of *Xcc* R1 resistance in linkage groups 1, 6, 8, and 9 (Iglesias-Bernabe et al., 2019).

In a previous study (Lu et al., 2021), we selected an *Xcc*-resistant line, Black rot Resistance 155 (BR155), and a susceptible line, SC31, by comparing symptom development. Using these two cabbage lines, we studied the early defense mechanisms of *B. oleracea* in response to *Xcc* infection and found that BR155 had a relatively strong antioxidant activity. These results suggest that regulating ROS accumulation during early *Xcc*–cabbage interactions may be essential for restricting symptom development. In this study, to identify QTLs for *Xcc* R1 resistance in BR155, we used a reference-based genotyping by sequencing (GBS) approach for single nucleotide polymorphism (SNP) identification and genotyping of a mapping population. The identified SNPs were used to construct linkage maps and to detect loci associated with black rot resistance.

## 2 Methods

### 2.1 Plant materials

In a previous study, two inbred cabbage lines (*Brassica oleracea L.* var. *capitate*), SC31 and BR155, showed susceptibility and high resistance to *Xcc* R1, respectively (Lu et al., 2021). In the current study, they were utilized as parents to generate a segregating population (F_1_, F_2_, and F_2:3_). A total of 126 F_2_ individuals were generated and self-pollinated to generate F_2:3_ progenies, and the F_2_ and F_2:3_ populations were used for genotyping and phenotypic evaluation, respectively. The parental lines and F_1_ and F_2_ plant materials examined in this study were obtained from Joeun Seeds Co. (Chungcheongbuk-Do, Korea), and F_2_ progenies were self-pollinated to produce seeds of F_2:3_ progenies in a greenhouse facility located at Chungnam National University.

### 2.2 Phenotypic screening and disease evaluation

*Xanthomonas campestris* pv. *campestris* KACC 10377 (*Xcc* R1) was used for the inoculation tests in this study, which was obtained from the Korean Agricultural Culture Collection (KACC; Suwon, Korea). The inoculum and inoculation protocol was conducted as described previously (Lee et al., 2020) with minor modification. Shortly, using an inoculating loop, the bacterial inoculum was streaked over tryptic soy agar (TSA) plates and incubated for 48 hours at 30°C. To prepare the bacterial solution for inoculation, cultivated bacteria were suspended in distilled water and diluted to an optical density (OD) of 0.125 at 600 nm. F_2:3_ seeds were sown and grown on 5×8-cell plug trays in a greenhouse. At 14–17 days after sowing, inoculation tests were performed until the plants had two completely expanded true leaves. The leaves were inoculated by spraying a bacterial suspension until the adaxial and abaxial surfaces of the leaves were sufficiently moistened. Subsequently, the inoculated plants were kept at a temperature of 28°C and high humidity for 48 h. Then, the temperature was adjusted to 25°C and other conditions were kept constant for a further 7 days of incubation, and the disease symptoms in two inoculated leaves of each plant were surveyed. The severity of the black rot symptoms was determined based on the infected leaf area using the following disease indexes: (0) no visible symptoms (immune I), (1) 1–25% infection (resistant, R), (2) 26–50% infection (moderately resistant MR), (3) 51–75% infection (moderately susceptible, MS), (4) 76–99% infection (susceptible, S), (5) 100% infection (highly susceptible, HS) (Peňázová et al., 2018).

### 2.3 Statistical analysis

SPSS software (v. 26.0, IBM, Armonk, NY, USA) was used for descriptive statistics and correlation analyses of each trial. The mean values of the three trials for each test were used to conduct correlation analyses. The coefficient of variation was calculated as σ/µ, where σ represents the standard deviation and µ represents the average.

### 2.4 GBS library preparation, sequencing, and SNP calling

To construct the GBS library for sequencing, the genomic DNAs of the parental lines and F_2_ population were isolated from their young leaf tissues via the modified CTAB method. The quantity and quality of the DNA were examined using a NanoDrop ND-1000 (Thermo Fisher Scientific Inc., USA) and agarose gel (1.5%) electrophoresis. For GBS analysis, a library was constructed using 128 genomic DNAs belonging to two parent lines and 126 F_2_ populations. The library construction was outsourced to SEEDERS sequencing company (Daejeon, Korea; http://www.seeders.co.kr/). Briefly, construction of the GBS library involved the following steps: adaptor annealing, digestion of genomic DNA using *ApeKI* (New England Biolabs, Ipswitch, MA, USA) restriction enzyme, pooling and purification of ligated products, and PCR amplification. The size distribution of the templates was confirmed by analyzing the PCR-enriched fragments on an Agilent Technologies 2100 Bioanalyzer with a DNA 1000 chip. The quality was then assessed through agarose gel electrophoresis. The barcode sequence was used to separate the raw sequences into individual samples, after which the adapter sequence was removed, trimming the sequence quality. We used cutadapt v.1.8.3 (Martin, 2011) for adapter trimming and the Dynamic Trim (phred score ≥ 20) and the LengthSort (short read length ≥ 25 bp) programs of the SolexaQA v.1.13 package for sequence quality trimming (Cox et al., 2010). To ensure accuracy, we used the consensus sequence of SC31 samples obtained through mapping on the corresponding reference genome (*Brassica oleracea*, v.2.1.28; EnsemblPlants, http://plants.ensembl.org/index.html) in resequencing as the reference sequence for analysis. This decision was made due to significant differences between the reference genome and the germplasm of the population being studied. Subsequently, the clean reads of each F2 individual were aligned to the SC31 consensus sequence reference sequence using the Burrows-Wheeler Aligner (BWA) program v.0.6.1-r104 (Li and Durbin, 2009). To create an SNP matrix, we compared the raw SNPs from 126 samples using SEEDERS’ in-house script (Kim et al., 2014). Subsequently, the SNPs were classified into homozygous (SNP read depth ≥ 90%), heterozygous (40% ≤ SNP read depth ≤ 60%), and others (homozygous/heterozygous; could not be distinguished by type), followed by SNP filtering (**Table S1**).

### 2.5 SNP genotyping and bin construction

Although GBS can rapidly detect thousands of SNPs, not all SNPs detected by GBS can be used to construct genetic maps for genotyping F2 populations. The noise present in sequencing reads can impact the construction of the linkage map. We conducted the chi-square (χ^2^) test on all SNPs to assess any potential segregation distortion, and SNPs with a segregation distortion test score of p < 0.05, or those with an abnormal base, were removed from the dataset. Additionally, any genotypes with more than 5% deletions were removed, along with corresponding individuals. Finally, we marked specific SNP positions that can be used for calling SNPs in F_2_ individuals. The genotype of F_2_ individuals was converted to 2 if the SNP was the same as SC31, the genotype of F_2_ individuals was converted to 0 if the SNP was the same as BR155, and the genotype of F_2_ individuals was converted to 0 if the SNP was the same as F_1_. A sliding-window approach was applied for variant calling errors to calculate the ratio of SNP alleles derived from the two parental lines, BR155 and SC31 (Huang et al., 2009). Genotypic data were scanned using a window size of 15 SNPs and a step size of 1. For each individual, the ratio of the SNP alleles from BR155 to SC31 within the window was calculated. Windows with a sum of 15 SNPs were greater than 24, which were considered from SC31, and less than 6, which were considered from BR155, whereas those with varied sums were classified as heterozygous. Adjacent windows with the same genotype were combined into blocks, and recombinant breakpoints were assumed to be at the boundaries of adjacent blocks with different genotypes. Next, a bin map was generated by aligning and comparing the genotypic maps of individual F_2_ plants. Consecutive intervals lacking a recombination event within the population were joined into bins that were used as markers. This process was performed using an R script.

### 2.6 Genetic map construction and QTL mapping

A linkage map was established from the recombination bins that were used as genetic markers via the JoinMap version 5.0 software (https://kyazma.nl/). The Kosambi mapping function was used to convert the recombination frequencies into genetic distances. The disease index for each F_2_ individual was calculated as the mean grade of 10–15 F_2:3_ seedlings. QTLs for *Xcc* resistance were evaluated using a composite interval mapping (CIM) analysis with WinQTL cartographer version 2.5 (Zeng, 1994; Rifkin, 2012).

### 2.7 RNA extraction and gene expression analysis by quantitative real-time PCR

The infected zones of the leaves were collected at 0, 12, 24, and 48 h after inoculation. For each time point, samples from five leaves were combined and considered biological replicates. The leaves were ground into a powder in liquid nitrogen. Total RNA was extracted using the RNeasy^®^ Plant Mini Kit (Qiagen, Hilden, Germany), following the manufacturer’s instructions. The extracted RNA was purified using RNeasy^®^ Plant Mini Columns (Qiagen, Hilden, Germany). cDNAs was synthesized using 1 μg of total RNA. A real-time PCR detection system (Bio-Rad, Hercules, CA, USA) with TB Green^®^ Premix Ex Taq ^®^ (TaKaRa Bio) was used to quantify the gene expression. Sequences of the gene-specific primers used for quantitative real-time PCR (qRT-PCR) are presented in **Supplementary Table S5**. The internal standard used was 18S rRNA. Each experiment was performed at least thrice. The 2^−ΔΔCt^ method was used to quantify the relative transcript level (Livak and Schmittgen, 2001).

3 Results

### 3.1 Evaluation of resistance to black rot

**Figure 1:**
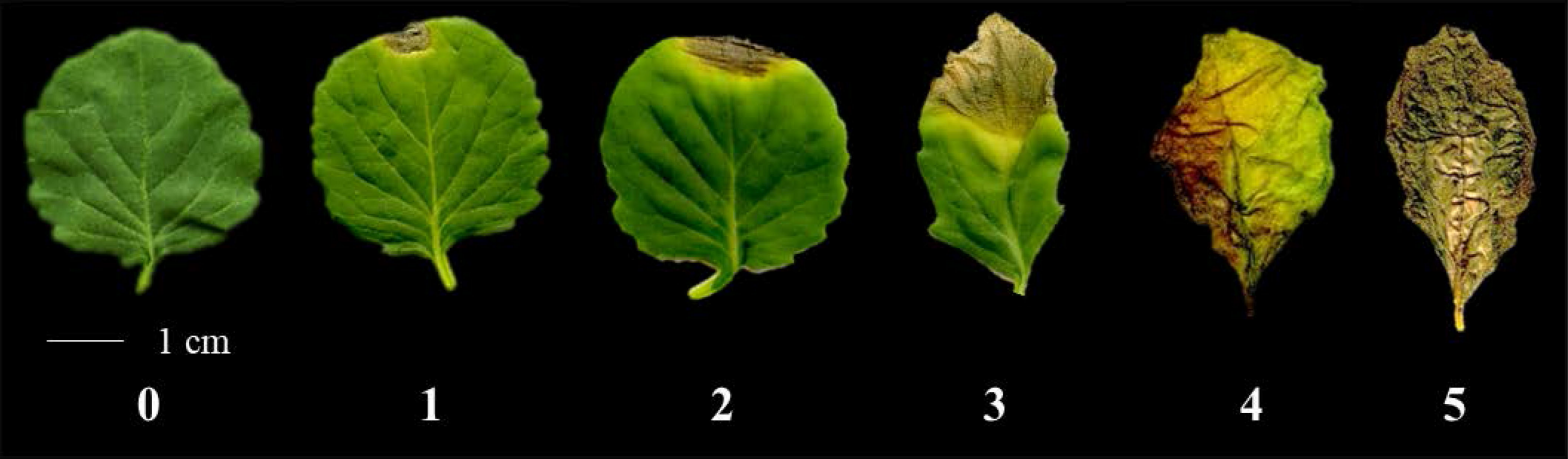
Representative black rot disease symptoms on leaves of cabbage after spraying with *Xcc* R1 suspension (OD_600 =_ 0.125). The severity of the black rot symptoms was recorded based on infected leaf area, with the following disease indices: (0) no visible symptoms, (1) 1–25% infection (resistant, R), (2) 26–50% infection (moderately resistant MR), (3) 51–75% infection (moderately susceptible, MS), (4) 76–99% infection (susceptible, S), (5) 100% infection (highly susceptible, HS).

**Figure 2:**
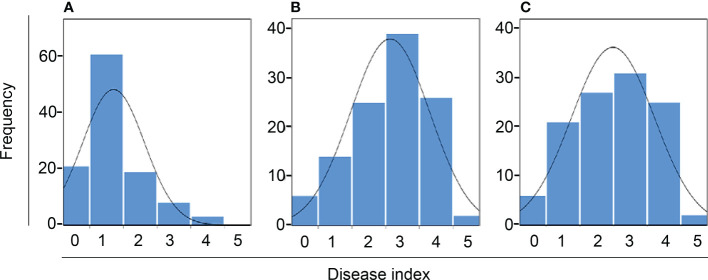
Frequency distribution of black rot disease index of F_2:3_. **(A–C)** represent the inoculation test result from summer 2020, fall 2020, and spring 2021, respectively. The curve indicates normal distribution.

We observed phenotypes 7 days after the *Xcc* R1 inoculation of parental lines (SC31 and BR155) and 126 F_2:3_ plants in three environments to evaluate black rot resistance (**Figure 1**). The average disease score from 10 to 15 plants for F_2:3_ plants was considered as the disease score of each F_2:3_ individual (**Table S2**). Inoculation tests were repeatedly carried out in the summer and fall of 2020, and in the spring of 2021 under the same conditions (**Figure 2**). The frequency distribution of the black rot disease index of the F_2:3_ population under the two different environmental conditions appeared to be approximately normally distributed in separate experiments, indicating that the black rot resistance phenotype was governed by multiple genes (**Figure 2**). The correlation coefficients among the three trials were calculated (**Table 1**). As a result of the correlation analysis between the three trials using 128 cabbage samples (parents and F_2:3_), the second and third trials had a positive correlation of approximately 0.66 at the level of α = 0.001 (^***^). On the other hand, the first and third trails had a negative correlation of roughly -0.20 (**Table 1**).

**Table 1:** Phenotypic correlation for black rot disease index over summer 2020, fall 2020, and spring 2021.

Trial	1^st^ (Summer 2020)	2^nd^ (Fall 2020)	3^rd^ (Spring 2021)
1^st^ (Summer 2020)	1		
2^nd^ (Fall 2020)	-0.13	1	
3^rd^ (Spring 2021)	-0.2	0.66***	1

***Significant at the 0.001 probability level.

### 3.2 Whole-genome resequencing of two cabbage parental lines and SNP detection by GBS

The whole-genome sequencing data contained 68 and 67 million raw reads for BR155 and SC31, respectively (**Table S3**). According to Parkin et al., the genome sequence for *B. oleracea* is 488.6 Mb, consisting of 446.9 Mb of nine pseudo-chromosomes and 41.2 Mb of unanchored scaffolds, accounting for approximately 75% of the estimated genome size of 648 Mb (Parkin et al., 2014). Our new sequencing data was approximately 24 times the genome size for both parent lines. We successfully mapped each set of paired reads onto the nine pseudo-chromosomes of the reference genome sequence. Of the raw reads obtained, 70.74% and 74.67% from BR155 and SC31, respectively, were successfully aligned to the reference genome, which resulted in a mapped region of 72.44% and 78.39% for BR155 and SC31, respectively (**Table S3**). The total number of SNPs and average SNP densities varied between the two parental lines. High-quality SNPs were identified in both BR155 and SC31, with approximately 1.02 million and 0.26 million SNPs, respectively. These SNPs were merged and used to detect SNPs between the two parental lines (**Table 2**).

**Table 2:** Distribution of SNPs on the *B. oleracea* genome.

Chr.	Chr. Length (bp)	No. genes^a^	Raw SNPs	Filtered SNP	Selected SNPs^b^
C01	43,764,888	5,401	44,427	1,940	528
C02	52,886,895	5,843	112,580	3,811	1,075
C03	64,984,695	8,490	89,494	3,618	1,040
C04	53,719,093	6,426	74,076	2,718	868
C05	46,902,585	5,851	71,841	2,931	828
C06	39,822,476	4,762	70,502	2,636	784
C07	48,366,697	5,752	94,037	3,645	1,030
C08	41,758,685	5,599	74,419	2,980	798
C09	54,679,868	6,642	76,718	3,124	989
Total	446,885,882	54,766	708,094	27,403	7,940

a Data source: EnsemblPlants (http://plants.ensembl.org/index.html).

b We removed F_2_ plants with > 25% missing and SNPs with missing in filtered SNPs.

**Figure 3:**
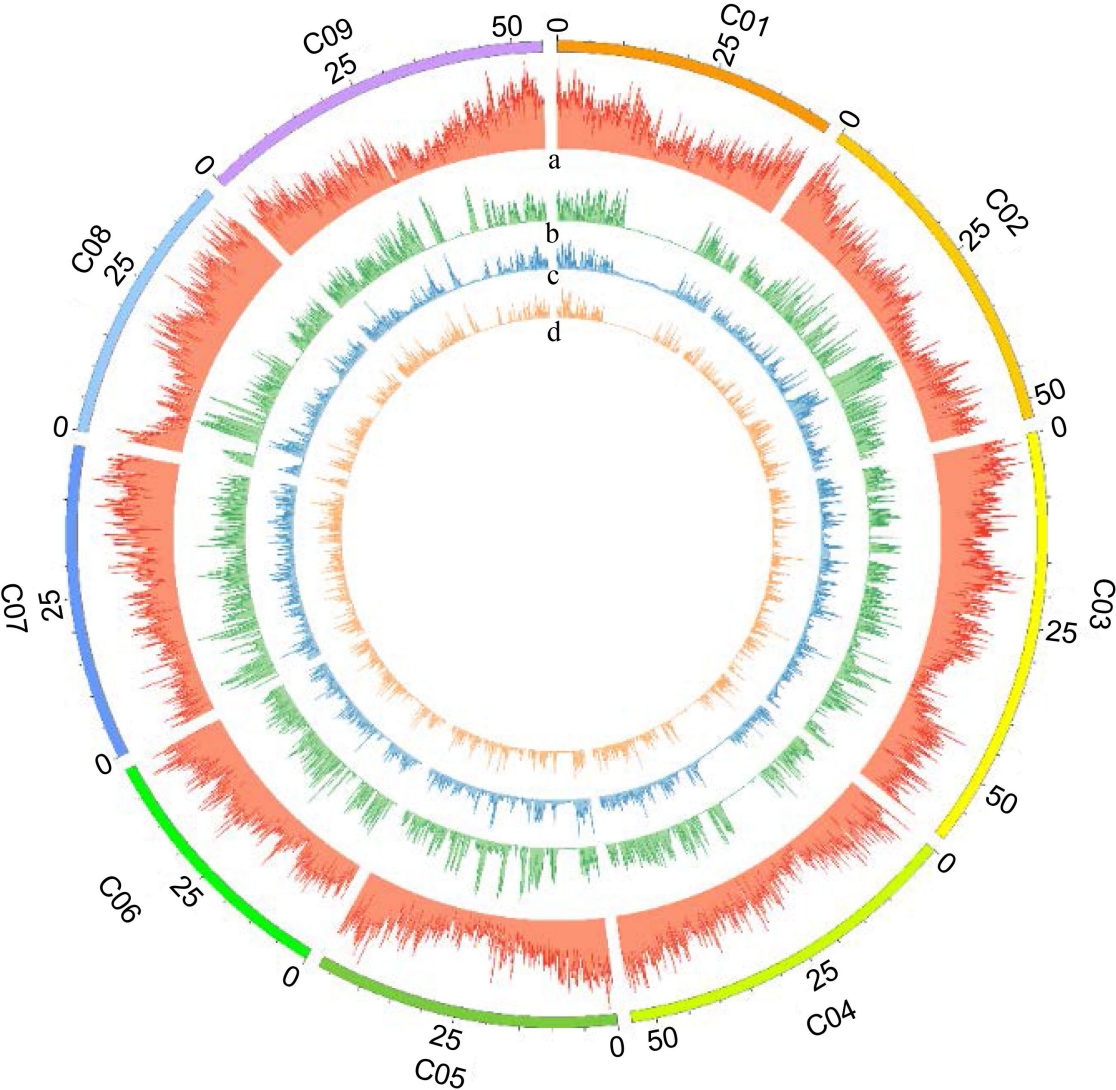
Distribution of identified SNPs on nine *B. oleracea* chromosomes. Distribution of SNPs detected on each *B. oleracea* chromosome (100 kb window size) was visualized using Circos diagram-Perl script. The outermost circle represents nine *B. oleracea* chromosomes designated as “C01-C09”. Panels as seen from outside to inside: (a) gene density, (b) raw SNPs, (c) filtered SNPs, and (d) selected SNPs for mapping on each chromosome.

For the genome-wide detection of SNPs in cabbage using GBS, the restriction enzyme *ApeKI* was used to digest genomic DNA and construct GBS libraries of the F_2_ plants and parents of the intraspecific mapping population (BR155 and SC31). Sequencing was performed on an Illumina high-throughput sequencing platform (Illumina HiSeq X sequencer), and a total of 1,501,825,142 raw sequence reads corresponding to 226.78 GB of sequence length were generated. The raw data contained an average of 97.11% of demultiplexed reads, with the overall GC content of the sequences being approximately 47.66%, And the Q30 score was approximately 91.29%. Raw SNPs were detected by sequence pre-processing and alignment to the SC31 consensus sequence, and a matrix containing 304,184 SNPs was obtained. The SC31 sequence was aligned to the *B. oleracea* (TO1000) sequence to determine the physical position of each SNP. Based on the filtering process, 27,403 polymorphic SNPs were identified in the cabbage F2 population (**Table 2**). SNPs were distributed across all nine *B. oleracea* chromosomes, as illustrated in **Figure 3** and **Table 3**.

**Table 3:** Distribution of bin markers on the cabbage genetic map.

Linkage group (Ch)	Bin No.	Genetic length (cM)	Average bins interval (cM)
C01	83	59.43	0.72
C02	97	64.67	0.67
C03	188	114.32	0.61
C04	110	87.90	0.80
C05	115	79.90	0.69
C06	87	49.02	0.56
C07	119	64.56	0.54
C08	96	60.55	0.63
C09	125	95.29	0.76
Total	1,020	675.64	0.66

### 3.3 Development of the linkage map for *Brassica oleracea*

**Figure 4:**
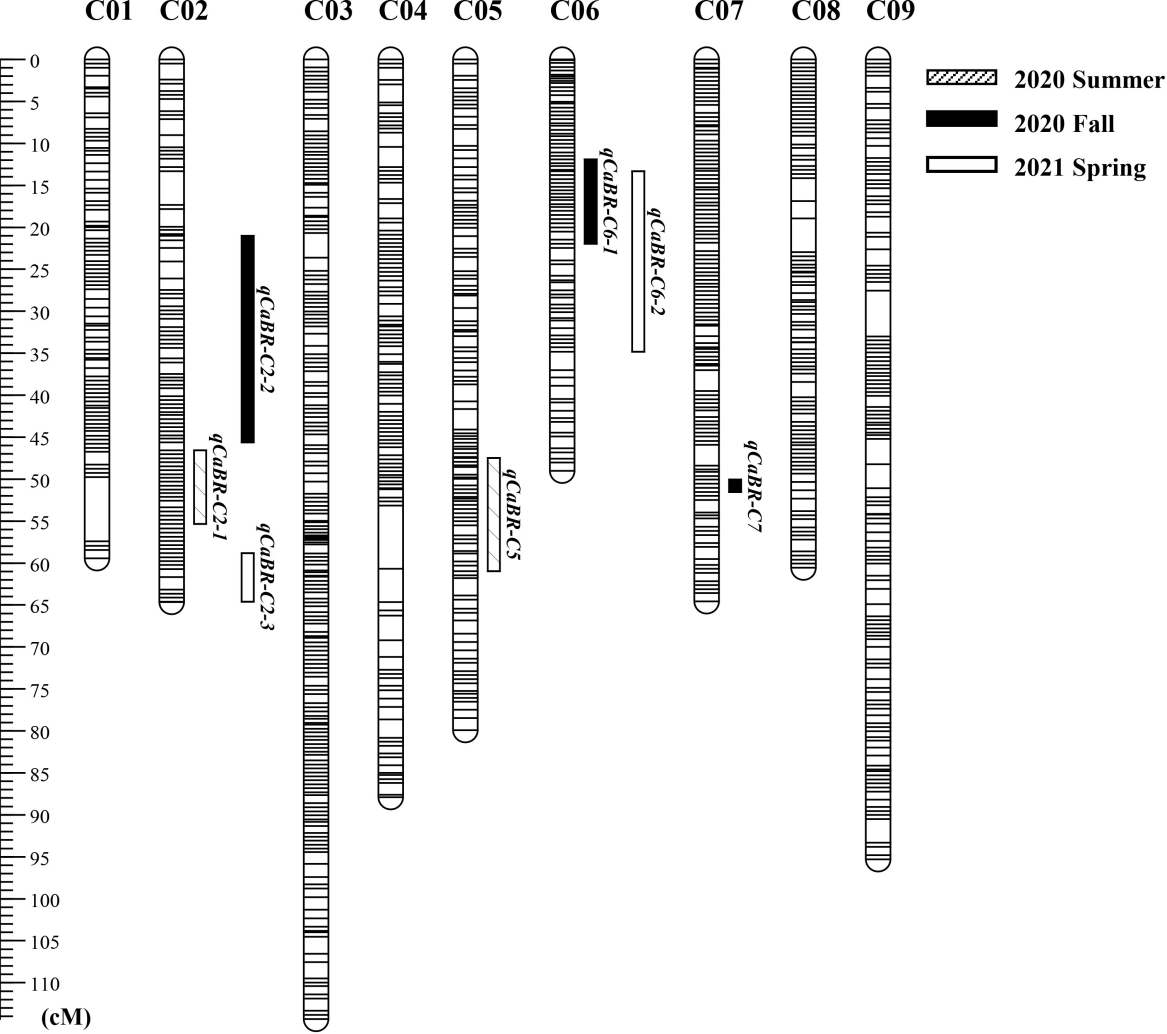
The genetic linkage map of cabbage constructed using the F_2_ lines derived from the parental lines SC31 × BR155. Detected QTLs were marked by rectangular bars with different fill in the right side of the linkage groups followed by QTL name.

After filtering the SNPs according to the genotyping criteria, 7,940 high-quality SNPs were identified between the two parents to generate bin markers for the F_2_ population (**Table 3**). A modified sliding window approach was adopted to determine the recombinant breakpoints for the F_2_ individuals. The adjacent bins of the same genotype were merged into identical bins. A high-density genetic linkage map was constructed using 1,020 recombination bins (**Figure 4**, **Table 3**). The total genetic distance of the linkage map was 675.64 cM with an average distance of 0.66 cM between adjacent bins. Linkage Group 3 (C03) contained the most bins (188), followed by Group C09 (125). Group 3 also comprised the longest linkage group, which spanned 114.32 cM and 95.29 cM with an average 0.61 cM and 0.76 cM marker intervals, respectively. The shortest linkage group was located at C06, which was 49.02 cM in length and harbored 87 bin markers with an average marker interval of 0.56 cM.

### 3.4 QTL analysis

Composite interval mapping was conducted using the developed cabbage map to detect black rot resistance QTL. QTL analysis was performed for each trial. QTLs were detected based on LOD scores higher than the threshold (2.0). As a result, a total of seven QTLs were detected in this study, the LOD values between 2.10 and 4.27 (**Figure 4**, **Table 4**; **Figure S1**). In the first test, performed in the summer of 2020, there were two significant QTL regions: *qCaBR-C2-1* on chromosome C02 and *qCaBR-C5* on chromosome C05 (**Figure 4**, **Table 4**; **Figure S1**). Among these, *qCaBR-C5* showed the highest LOD score, 4.27 (**Figure 4**, **Table 4**; **Figure S1**). In fall 2020, the second test identified three QTLs, *qCaBR-C2-2*, *qCaBR-C6-1*, and *qCaBR-C7* on chromosomes C02, C06, and C07, respectively. Two QTLs were detected in spring 2021: *qCaBR-C2-3* and *qCaBR-C6-2*.

**Table 4:** Details of resistance QTLs related *Xcc* R1.

Inoculation test	QTL name	Linkage group	Number of bin	Position (cM)	Physical position (bp)	LOD^a^	Add^b^	Dominance effect	R^2^ (%)^c^
(1^st^) 2020 Summer	*qCaBR-C2-1*	C02	19	46.57 - 54.86	16,499,782-49,025,692	2.55	0.37	-0.25	8.2
	*qCaBR-C5*	C05	27	47.47 - 60.98	12,846,418-42,204,163	4.27	-0.46	-0.45	13.6
(2^nd^) 2020 Fall	*qCaBR-C2-2*	C02	38	21.02 - 45.62	3,513,666-16,499,781	3.42	0.56	0.19	12.2
	*qCaBR-C6-1*	C06	23	11.93 - 21.96	27,081,550-34,373,426	2.18	-0.27	-0.51	7.28
	*qCaBR-C7*	C07	4	50.05 - 51.55	44,486,293-45,629,195	2.1	0.4	0.33	6.75
(3^rd^) 2021 Spring	*qCaBR-C2-3*	C02	6	59.30 - 63.15	50,467,819-52,028,484	2.39	-0.22	0.65	8.03
	*qCaBR-C6-2*	C06	39	13.32 - 34.84	29,853,043-37,960,957	2.76	-0.56	-0.38	9.9

a Logarithm of odds ratio at the position of the peak.

b Additive effect of QTL.

c Percent of phenotypic variance explained by the QTL.

### 3.5 Prediction of candidate genes for black rot resistance in *B. oleracea*

**Figure 5:**
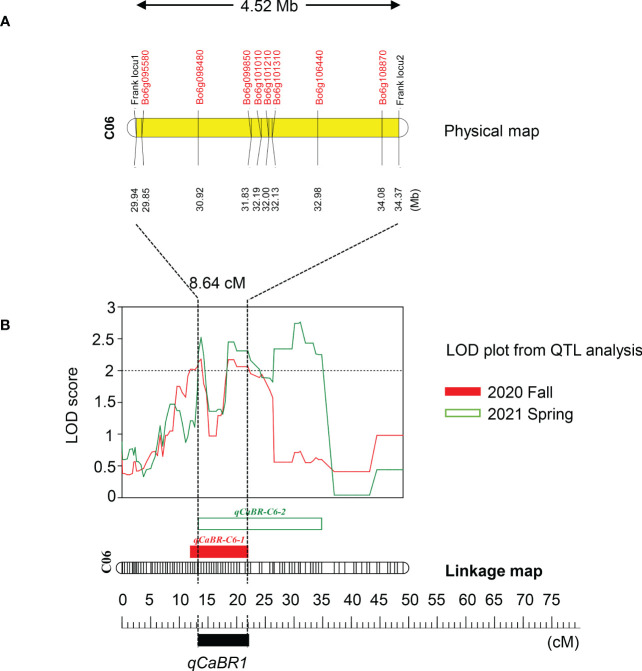
Map of the corresponding qCaBR1 region of the B. oleracea genome. **(A)** The corresponding physical map showed the location of the major QTL region in the B. oleracea reference genome and the included reference genes associated with biotic stresses. **(B)** The major QTL was named qCaBR1 as the overlapping section, detected on linkage group six in fall 2020 and spring 2021, respectively. The genetic position of QTLs were indicated in centimorgans (cM) and marked by rectangular bars with a different color in the left side of the linkage group followed by the QTL name. The change curves of LOD values obtained from the results of the three inoculation experiments were shown in red, green, and yellow for fall 2020, spring 2021, and the overlapping section, respectively.

**Figure 6:**
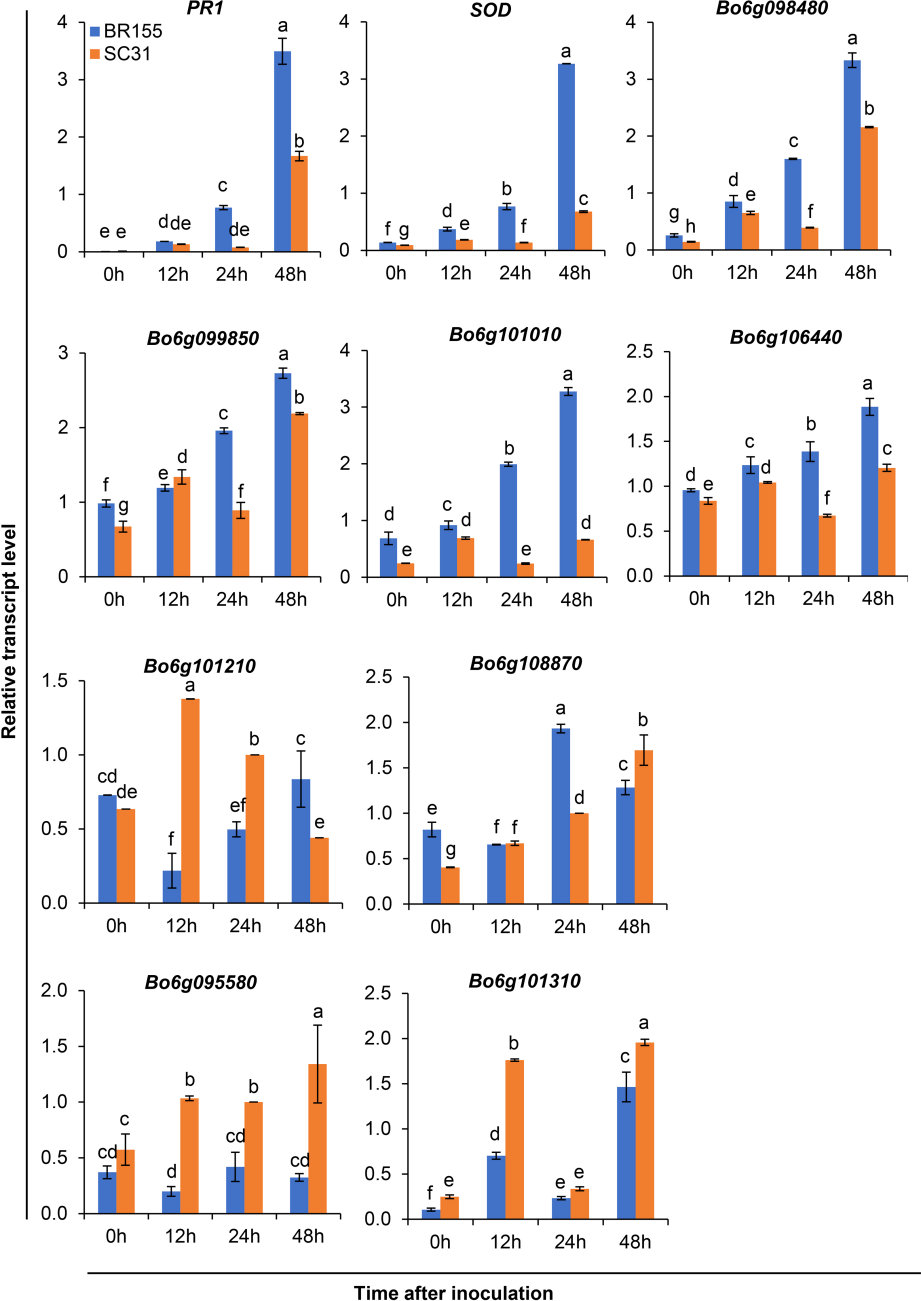
Relative transcript levels of defense-related genes (PR1 and SOD) and eight candidate genes in leaves of resistant (BR155) and susceptible (SC31) cabbage lines. Gene expression level was determined by qRT-PCR at the indicated time after Xcc R1 inoculation and normalized to transcript levels of the 18S rRNA gene. Error bars represent standard deviations of three replicates. Similar results were obtained in at least two independent experiments. Different letters indicate significant differences among samples (a = 0.05, one-way ANOVA and Duncan’s multiple range test).

Based on the above analysis, among all seven QTLs (**Figure 4**, **Table 5**), we found overlapping regions between the second and third trials located on chromosome C06 which were designated as the major QTL loci. The major QTL identified on chromosome 6 was *qCaBR1* (Cabbage Black rot Resistance-1) (**Figure 5**). According to the available *B. oleracea* (TO1000) genome sequence (http://plants.ensembl.org/index.html), *qCaBR1* was detected on C06:29,853,043-34,373,426 (4.52 Mb) (**Figure 5**, **Table 5**) and included 591 genes. Of the 591 *B. oleracea* genes, 96 (**Table S4**) had putative gene annotation data (Arabidopsis orthologs), for which we could categorize the functional groups (https://www.arabidopsis.org). According to the annotation information, eight out of 96 genes responded to biotic stimuli and were related to defense responses against other organisms (bacteria, fungi, and oomycetes) (**Table 6**). We compared the expression patterns of eight candidate genes (**Figure 6**) that may be related to black rot resistance after *Xcc* R1 inoculation (12, 24, and 48 h) in the two cabbage lines, BR155 and SC31, to assess their defense-related responses. In BR155, two defense-related genes, *PR1* and *SOD*, were rapidly and strongly induced by *Xcc* inoculation, and thus can be used as early defense response markers (Lu et al., 2021). In this study, we also observed that the *Xcc*-induced expression levels of *PR1* and *SOD* was more than two times higher than that in BR155 plants when compared to SC31 plants. A high level of relative expression was observed for four genes (Bo6g098480, Bo6g099850, Bo6g101010, and Bo6g106440) at all time points in BR155; these results were similar to those of *PR1* and *SOD*. However, the expression patterns of Bo6g095580 and Bo6g101310 were opposite those of *PR1* and *SOD*. As shown in **Figure 6**, the *Xcc*-induced expression levels of Bo6g095580, Bo6g101310, and Bo6g101210 were higher in the susceptible line SC31 than in BR155 (resistant line). Interestingly, the gene expression pattern of Bo6g108870 significantly increased only 24 h post-inoculation in BR155, a resistant parental line (**Figure 6**). These eight genes showed differential expression patterns between the BR155 and SC31 plants in response to *Xcc* inoculation. Thus, the qRT-PCR results indicate that all eight genes selected from the major QTL interval may be involved in the black rot resistance of BR155.

**Table 5:** The physical position of major QTL related to black rot resistance in *B. oleracea*.

QTL name	QTL position in linkage group (cM)	Physical position (bp)	A	B	C
*qCaBR1*	C06:13.32 - 21.96	C06: 29,853,043 - 34,373,426	591	96	8

A, Number of predicted genes gene in QTL region.

B, Number of genes with functional annotation in QTL region.

C, Number of genes associated with response to biotic stimulus in QTL region.

**Table 6:** A list of genes associated with response to biotic stimulus located in the major QTL interval.

B. oleracea_ID	Arabidopsis_ID	Description	Response to biotic stimulus	References
Bo6g095580	AT1G69370	Chorismate mutase 3	defense response to bacterium	Mobley et al., 1999
Bo6g098480	AT1G67880	ß-1,4-N-acetylglucosaminyltransferase family protein	defense response to bacterium, fungus	Depuydt and Vandepoele, 2021
Bo6g099850	AT1G66340	Ethylene receptor 1	defense response to bacterium	Clay et al., 2009
Bo6g101010	AT1G66480	Plastid movement impaired 2	response to fungus	Didelon et al., 2020
Bo6g101210	AT1G66730	DNA ligase 6	response to molecule of bacterial origin	Park et al., 2015
Bo6g101310	AT1G66830	Leucine-rich repeat protein kinase family protein	defense response to bacterium, fungus	Depuydt and Vandepoele, 2021
Bo6g106440	AT1G67950	RNA-binding family protein	defense response to fungus	Li et al., 2019
Bo6g108870	AT1G69450	Early-responsive to dehydration stress protein	defense response to other organism	Depuydt and Vandepoele, 2021

## 3 Discussion

The resistant line “BR155” and susceptible line “SC31” used in this study were selected from 94 *B. oleracea* lines by comparing the lesion areas after pathogenicity assays using the scissor-clipping method (Lu et al., 2021). SC31 was one of 23 lines with a symptom area of 90% or more, and BR155 was the most resistant cabbage line with a lesion area of <10%. Previous studies have indicated that BR155 may carry a highly effective resistance gene or locus. We compared the two cabbage lines for the *Xcc*-induced expression pattern of 13 defense-related genes. Among them, the *Xcc*-induced expression levels of *PR1* and antioxidant-related genes (*SOD*, *POD*, *APX*, *Trx H*, and *CHI*) in BR155 were over twice as high as those in SC31. Nitroblue tetrazolium (NBT) and diaminobenzidine tetrahydrochloride (DAB) staining analysis showed that BR155 accumulated less *Xcc*-induced reactive oxygen species (ROS) than did SC31. Furthermore, 2, 2-diphenyl-1-picrylhydrazyl (DPPH) radical scavenging assays showed that BR155 had higher antioxidant activity than SC31 (Lu et al., 2021). Identifying the resistance locus of BR155 will be crucial for understanding the mechanism of black rot disease resistance. Therefore, in the present study, a GBS-based genetic linkage map was developed and QTL linked to resistance against *Xcc* R1 in cabbage was identified.

Genotyping-by-sequencing (GBS) technology allows for efficient and cost-effective genotyping of large numbers of markers across the genome. Here, we applied high-throughput GBS technology with the type-II restriction endonuclease *ApeK*I (Elshire et al., 2011) to the F_2_ group of cabbage, enabling the simultaneous identification of sufficient polymorphic SNPs and genotyping. This allowed us to create a linkage map with reasonably high density without the need to check or apply existing markers (**Figure 4**). A modified sliding window approach was adopted to determine the recombinant breakpoints for the F_2_ individuals. The sliding-window approach involves calculating the recombination frequency between adjacent markers within each window. Recombination frequency measures how often genetic recombination occurs between two markers during meiosis, and can be used to estimate the physical distance between them on the chromosome (Tang et al., 2009; Beissinger et al., 2015). The GBS analysis performed by Parkin et al. identified 826 bins in *B. oleracea* (Parkin et al., 2014), which was fewer than that identified in this study (1020 bins). Our higher bin numbers are probably due to the difference in the genetic diversity of the parental lines and the number of segregating progenies used for GBS analysis. We used BR155 and SC31 as parental lines, which have variable genetic diversity, whereas Parkin et al. analyzed the population of double haploid (DH) kale-like and DH broccoli lines. We also analyzed 126 F_2_ plants for mapping. In this study, we identified 708,094 SNPs between BR155 and SC31 using 24x genome coverage whole-genome resequencing (**Tables 2**; **S3**). Here, 27,403 GBS-based SNPs were detected between the parental lines, which was 7-fold fewer than those detected by resequencing. After filtering the SNPs according to the genotyping criteria, 7,940 high-quality SNPs were identified between the two parents to generate bin markers for the F_2_ population (**Table 2**). The total genetic distance of the linkage map was 675.64 cM, with an average length of 0.66 cM between adjacent bins (**Table 3**).

We evaluated black rot resistance by observing phenotypes seven days after inoculating parental lines (SC31 and BR155) and 126 F_2:3_ plants with *Xcc* R1. The inoculation tests were performed three times throughout the summer and fall of 2020, as well as in the spring of 2021, all under identical conditions (**Figure 2**). As shown in **Table 1**, analysis of the correlation between three trials consisting of 128 cabbage samples, including parents and F_2:3_, revealed that the second and third trials had a strong positive correlation of approximately 0.66 at a significance level of α = 0.001 (***). In contrast, the first and third trials displayed a negative correlation of approximately -0.20. It seems possible that such an unexpected result in the first trials’ outcome was affected by the high temperature during summer. In 1972, Staub and Williams analyzed the impact of temperature on the black rot resistance of cabbage by exposing resistant and susceptible cabbage varieties inoculated with *Xcc* to various temperatures and analyzing the severity of black rot. Their results showed that although the traits of the resistant cabbage were evident at 20-24°C, the resistant cabbage was just as susceptible to the disease as the susceptible ones at 28°C (Staub, 1972). In Korea, the temperature inside a greenhouse can exceed 30°C during the summer.

*Xcc* exhibits high genetic diversity, and 11 races have been discovered worldwide. Among these races, R1 and R4 are the most prevalent and highly virulent among many commercial cultivars (Vicente et al., 2001; Cruz et al., 2017). Only a few resistant resources have been identified recently, considerably challenging the breeding of resistant cabbage cultivars. Several studies have identified R-genes/QTLs and markers associated with *Xcc* R1 and *Xcc* R4 resistance in *B. oleracea* (Camargo et al., 1995; Kifuji et al., 2012; Tonu et al., 2013; Saha et al., 2014b; Lee et al., 2015; Iglesias-Bernabe et al., 2019). In total, more than 15 QTLs were identified on *the B. oleracea* chromosomes, indicating that resistance to black rot is highly complicated. We identified seven QTLs related to the resistance to R1 of *Xcc* on C02, C05, C06, and C07. Since resistance was quantitative and under polygenic control, we confirmed the results of other studies. The positions of our black rot resistance QTLs coincided with those previously reported (Kifuji et al., 2012; Tonu et al., 2013; Saha et al., 2014b; Iglesias-Bernabe et al., 2019), except for QTL *qCaBR-*C7, which may represent a novel variation. Among the seven QTLs identified in this study, *qCaBR-C6-1* and *qCaBR-C6-2* were detected repeatedly in the two independent inoculation tests, had high LOD values, and accounted for a high percentage of the variation in all trials. We designated the overlapping part of these two QTLs on C06 as *qCaBR1* QTL. In addition, it was a strong candidate as a major QTL for black rot resistance. Regarding physical location, the major QTL, *qCaBR1*, found in our work is likely related to *BRQTL-C6* (Lee et al., 2015) and *Xcc*6.1 (Iglesias-Bernabe et al., 2019). Afrin selected five markers capable of distinguishing the resistant lines from the susceptible ones of cabbage consistently (Afrin et al., 2018). The SSR marker OI10G06 is one of these five markers. Interestingly, OI10G06 is located on chromosome C06 (C6:29898028-29898121) and is in the major QTL *qCaBR1* (C06:29,853,043–34,373,426) (**Table 5**). In the case of *BRQTL-C6*, the exact *Xcc* race used in the study is yet to be classified, and OI10G06 was able to separate resistant and susceptible lines but did not perfectly match the phenotypic data. However, these repeated reports related to *Xcc* resistance QTL strongly, supporting the idea that the QTL *qCaBR1* is involved in resistance to *Xcc* R1.

The information on the QTLs identified in this study will assist in the understanding of the molecular mechanisms of disease response in *B. oleracea* under *Xcc* stress. According to the available *B. oleracea* genome sequence (http://plants.ensembl.org/), the *qCaBR1* locus was delimited to a 4.53-Mb genomic region, which included 96 functionally annotated Arabidopsis orthologs. We identified candidate genes within this chromosomal region with the FGENESH online program (http://linux1.softberry.com/berry.phtml), and the NCBI BLASTP algorithm (http://blast.ncbi.nlm.nih.g/blast) (**Table S4**). We also identified gene ontology terms using the “Go Term Enrichment tool” on the Tair home page (https://www.arabidopsis.org/tools/go_term_enrichment.jsp). According to this analysis, eight of the 96 genes were biotic-stimulus-responsive (**Table 6**). These genes are involved in the resistance responses to bacterial or fungal diseases (Mobley et al., 1999; Clay et al., 2009; Li et al., 2019; Depuydt and Vandepoele, 2021) and microbe-associated molecular patterns (Park et al., 2015; Didelon et al., 2020). Since these genes were also speculated to play a role in defense responses against invading pathogens in cabbage, we designated them as candidate genes associated with black rot resistance in the BR155 line. To further assess their roles in black rot resistance, we performed an expression analysis of eight candidate genes (**Figure 6**). As expected, most of the candidate genes tested showed more robust expression than in SC31 from BR155 cells, a resistant line. However, in the case of Bo6g095580 and Bo6g101310, their expression was more strongly induced in SC31, a susceptible line, in response to *Xcc* inoculation. In the case of Bo6g101210, the *Xcc*-induced expression level was initially high in the susceptible lines; however, after 48 h, it increased in the resistant lines (**Figure 6**). Bo6g098480, Bo6g101310, and Bo6g108870 showed high homology to AT1G67880, AT1G66830, and AT1G69450, respectively. Depuydt and Vandepoele (2021) inferred the functions of several unknown Arabidopsis genes through omics-supported functional annotation analysis and classified AT1G67880, AT1G66830, and AT1G69450 as having functions related to plant disease resistance (Depuydt and Vandepoele, 2021). Bo6g099850 showed high homology with the Arabidopsis ethylene receptor 1 (AT1G66340). AtETR1, an ET receptor, is required for ET perception (Schaller and Bleecker, 1995) and for the microbe-associated molecular pattern (MAMP)-triggered immune response (MTI). A previous study found that etr1-1 and etr1-3 mutants of the ET signaling pathway were impaired in the Flg22-induced callose response, an MTI (Clay et al., 2009). Bo6g101010 showed a high homology of AT1G66480 to Arabidopsis. AT1G66480 is a protein with pathogen and abiotic stress responses, cadmium tolerance, and disordered region-containing (PADRE) domains. Moreover, the latter is in the top 10% of most induced genes after infection with the fungal pathogen *Sclerotinia sclerotiorum* (Didelon et al., 2020). Bo6g106440 shows high homology with Arabidopsis BPL2 (AT1G67950). Arabidopsis accelerated cell death11 (ACD11) encodes a sphingosine transfer protein, and knockout of ACD11 activates PCD and defense responses (Brodersen et al., 2002). BPA1 (the binding partner of ACD11) and its close homologs (BPLs, BPA1-Like proteins) are novel regulators controlling the ROS-mediated defense response and are targeted and manipulated by a virulence effector of Phytophthora, RxLR207. RxLR207 promotes pathogen infection by binding and degrading BPA1 and BPLs (Li et al., 2019). Bo6g095580 had high homology with Arabidopsis Chorismate mutase 3 (AtCM3; AT1G69370). CMs are enzymes that catalyze the conversion of chorismate, a key intermediate in the shikimate pathway, to prephenate, a precursor of the aromatic amino acids phenylalanine and tyrosine (Eberhard et al., 1996). In Arabidopsis, two plastid-localized CMs are allosterically regulated (AtCM1 and AtCM3) and one cytosolic isoform (AtCM2) is unregulated (Eberhard et al., 1996; Mobley et al., 1999). AtCM3-like isoforms are found only in the Brassicaceae family, suggesting that AtCM3-like isoforms may play a role in specialized metabolite production and stress responses in Brassicaceae (Westfall et al., 2014). For example, indole glucosinolates (IGs) are plant secondary metabolites derived from the amino acid tryptophan found in the family Brassicaceae, and IG synthesis requires indole- and sulfur-containing amino acids and activation of AtCM3 (Grubb and Abel, 2006). Bo6g101210 shares high homology with AT1G66730 in Arabidopsis. AT1G66730 encodes a novel plant-specific DNA ligase, DNA LIGASE VI, which is involved in the response to molecules of bacterial origin (Park et al., 2015). Expression analysis of these eight candidate genes in major QTL intervals and their functional characterization may provide additional molecular information regarding the role of this genomic region in controlling *Xcc* R1 resistance in *B. oleracea*.

In this study, we identified SNPs in the *B. oleracea* genome using a GBS approach. Using the identified SNPs, a linkage map of BR155 and SC31 was constructed. In addition, we mapped one major QTL and seven minor QTLs for *Xcc* R1 resistance. The information generated on QTLs is useful for fine mapping and future MAS of traits. In addition, these results can be applied to the development *Xcc* R1-resistant genotypes and the molecular dissection of *Xcc* R1 resistance in *B. oleracea.*

## Data availability statement

The datasets presented in this study can be found in online repositories. The name of the repository and accession number can be found below: NCBI; PRJNA974155.

## Author contributions

SY: Data curation. SY: Funding acquisition. LL and SY: Investigation. SC, LL and SY: Methodology. SY: Project administration. YL and S-YK: Resources. LL and SY: Visualization. LL, SY, and S-YK: Manuscript writing. All authors contributed to the article and approved the submitted version.
