# Supplementary figures and images for: A GBS-based genetic linkage map and quantitative trait loci (QTL) associated with resistance to Xanthomonas campestris pv. campestris race 1 identified in Brassica oleracea
Source: Front Plant Sci. 2023 Jun 13;14:1205681. doi: 10.3389/fpls.2023.1205681 (PMC10293835; doi:10.3389/fpls.2023.1205681)

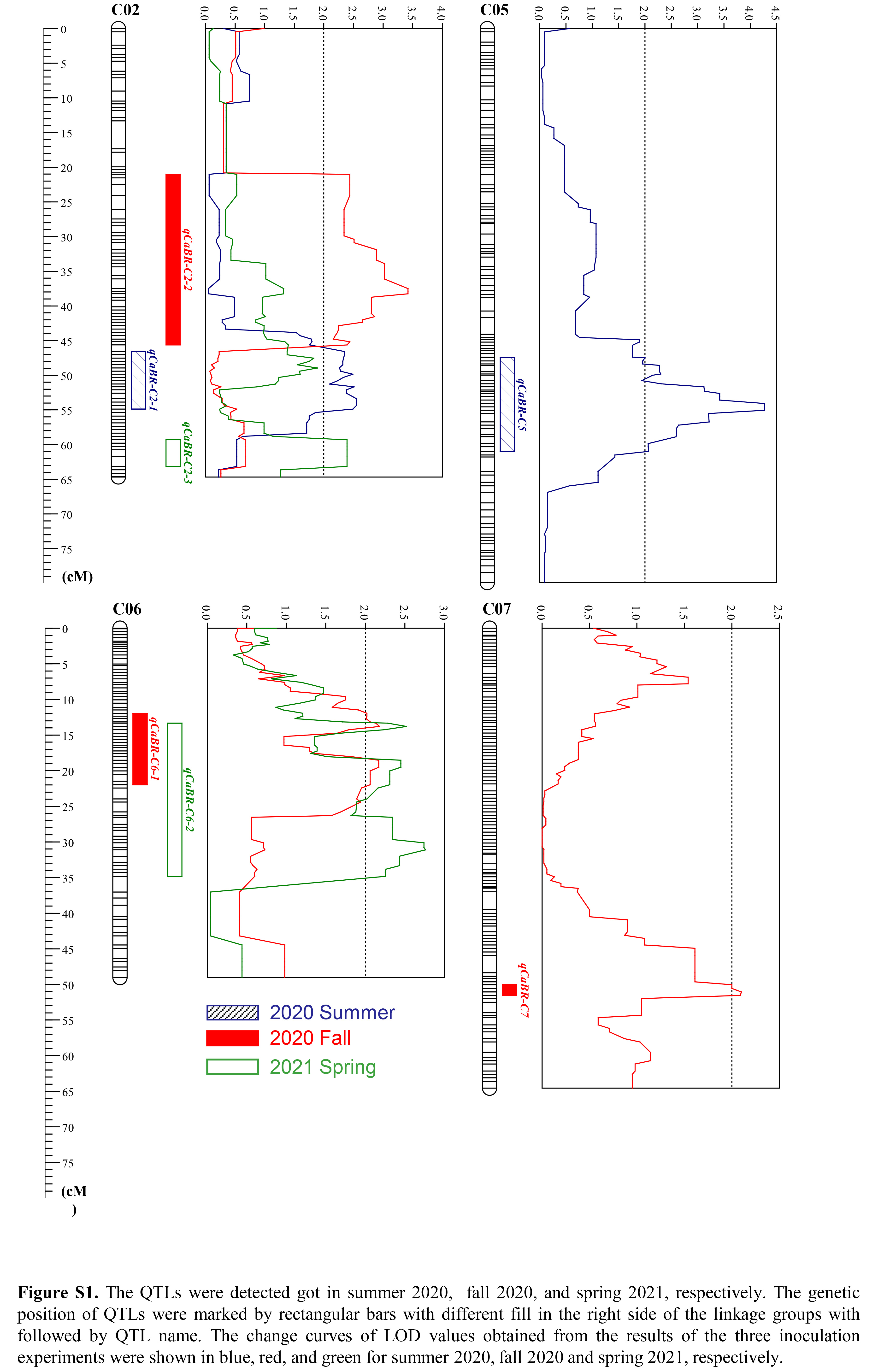

Supplement: Supplementary file 1 [file Image_1.jpeg]
