# Supplementary material for: A GBS-based genetic linkage map and quantitative trait loci (QTL) associated with resistance to Xanthomonas campestris pv. campestris race 1 identified in Brassica oleracea
Source: Front Plant Sci. 2023 Jun 13;14:1205681. doi: 10.3389/fpls.2023.1205681 (PMC10293835; doi:10.3389/fpls.2023.1205681)
Supplement: Supplementary file 2 [file Table_1.docx]

**Table S1.** Summary of SNP filter process.

| **Filter stage** | **Filter** | **SNP matrix loci** | |
| --- | --- | --- | --- |
| 1 | Total SNP matrix | 304,184 | |
| 2 | Parental lines Reseq. Polymorphic homo-type and consistent | 62,829 | |
| 3 | MAF (minor allele frequency) > 5%^a^ | 55,278 | |
| 4 | Missing data < 30%^b^ | 30,007 | |
| 5 | MAF > 5% and Missing data < 30% | 28,258 | |
| 6 | 100% homo-type | 27,403 | |
| ^a^MAF (minor allele frequency) >5%: SNPs with a minor allele frequency greater than 5% were selected. | | |  |
| ^b^Missing data <30%: SNPs with less than 30% missing data are selected. | | |  |
