# Supplementary material for: A GBS-based genetic linkage map and quantitative trait loci (QTL) associated with resistance to Xanthomonas campestris pv. campestris race 1 identified in Brassica oleracea
Source: Front Plant Sci. 2023 Jun 13;14:1205681. doi: 10.3389/fpls.2023.1205681 (PMC10293835; doi:10.3389/fpls.2023.1205681)
Supplement: Supplementary file 3 [file Table_2.docx]

**Table S2.** The average disease score of parental lines and each F_2:3_ individual against *Xcc* R1 in three different seasons.

| **1st trial_2020 summer** | |  | **2^nd^ trial_2020 fall** | |  | **3^rd^ trial_2021_spring** | |
| --- | --- | --- | --- | --- | --- | --- | --- |
| **Lines** | **average score (n>10)** |  | **Lines** | **average score (n>10)** |  | **Lines** | **average score (n>10)** |
| BR155 | 30 |  | BR155 | 1.5 |  | BR155 | 4.5 |
| SC31 | 57.5 |  | SC31 | 85.5 |  | SC31 | 60 |
| Combi1 | 22.5 |  | Combi1 | 57.5 |  | Combi1 | 22 |
| CaBR_3 | 0 |  | CaBR_3 | 5.5 |  | CaBR_3 | 0 |
| CaBR_9 | 47.2 |  | CaBR_9 | 84.5 |  | CaBR_9 | 39 |
| CaBR_11 | 13.6 |  | CaBR_11 | 56.1 |  | CaBR_11 | 13.5 |
| CaBR_15 | 52.2 |  | CaBR_15 | 91.5 |  | CaBR_15 | 51.5 |
| CaBR_17 | 22.5 |  | CaBR_17 | 59.5 |  | CaBR_17 | 38.3 |
| CaBR_19 | 43 |  | CaBR_19 | 67.5 |  | CaBR_19 | 21 |
| CaBR_20 | 11 |  | CaBR_20 | 65.5 |  | CaBR_20 | 14.5 |
| CaBR_21 | 27 |  | CaBR_21 | 90 |  | CaBR_21 | 3 |
| CaBR_23 | 0 |  | CaBR_23 | 95.5 |  | CaBR_23 | 97 |
| CaBR_30 | 10 |  | CaBR_30 | 71.4 |  | CaBR_30 | 65.6 |
| CaBR_32 | 1 |  | CaBR_32 | 62.5 |  | CaBR_32 | 28.3 |
| CaBR_33 | 11.1 |  | CaBR_33 | 5.6 |  | CaBR_33 | 62.1 |
| CaBR_35 | 3.5 |  | CaBR_35 | 20 |  | CaBR_35 | 17 |
| CaBR_38 | 0 |  | CaBR_38 | 77.5 |  | CaBR_38 | 41 |
| CaBR_43 | 0.6 |  | CaBR_43 | 100 |  | CaBR_43 | 100 |
| CaBR_44 | 6.3 |  | CaBR_44 | 76 |  | CaBR_44 | 49.5 |
| CaBR_45 | 5 |  | CaBR_45 | 89.5 |  | CaBR_45 | 26 |
| CaBR_46 | 13.3 |  | CaBR_46 | 45 |  | CaBR_46 | 0 |
| CaBR_47 | 10.5 |  | CaBR_47 | 82 |  | CaBR_47 | 66 |
| CaBR_49 | 0 |  | CaBR_49 | 50.5 |  | CaBR_49 | 0 |
| CaBR_50 | 12.8 |  | CaBR_50 | 43.5 |  | CaBR_50 | 7.5 |
| CaBR_52 | 8 |  | CaBR_52 | 16 |  | CaBR_52 | 7 |
| CaBR_53 | 29 |  | CaBR_53 | 29.5 |  | CaBR_53 | 34 |
| CaBR_54 | 49.5 |  | CaBR_54 | 61.5 |  | CaBR_54 | 92.5 |
| CaBR_55 | 5.5 |  | CaBR_55 | 28 |  | CaBR_55 | 7.5 |
| CaBR_56 | 14 |  | CaBR_56 | 41.7 |  | CaBR_56 | 35.5 |
| CaBR_57 | 13.8 |  | CaBR_57 | 52 |  | CaBR_57 | 38.5 |
| CaBR_59 | 42 |  | CaBR_59 | 45 |  | CaBR_59 | 47 |
| CaBR_60 | 3 |  | CaBR_60 | 44 |  | CaBR_60 | 28 |
| CaBR_61 | 15.6 |  | CaBR_61 | 84 |  | CaBR_61 | 93 |
| CaBR_63 | 1.5 |  | CaBR_63 | 77.5 |  | CaBR_63 | 49.5 |
| CaBR_64 | 18 |  | CaBR_64 | 87 |  | CaBR_64 | 45 |
| CaBR_65 | 0.6 |  | CaBR_65 | 63.5 |  | CaBR_65 | 90.7 |
| CaBR_67 | 20 |  | CaBR_67 | 20.5 |  | CaBR_67 | 3.5 |
| CaBR_68 | 12.2 |  | CaBR_68 | 78 |  | CaBR_68 | 76 |
| CaBR_74 | 12.8 |  | CaBR_74 | 88.9 |  | CaBR_74 | 80 |
| CaBR_75 | 3.8 |  | CaBR_75 | 94.4 |  | CaBR_75 | 68 |
| CaBR_76 | 0 |  | CaBR_76 | 60.5 |  | CaBR_76 | 80 |
| CaBR_77 | 5 |  | CaBR_77 | 80.5 |  | CaBR_77 | 69.5 |
| CaBR_78 | 10 |  | CaBR_78 | 86 |  | CaBR_78 | 86 |
| CaBR_79 | 0 |  | CaBR_79 | 91.7 |  | CaBR_79 | 97.8 |
| CaBR_81 | 0 |  | CaBR_81 | 80 |  | CaBR_81 | 88.9 |
| CaBR_82 | 37.9 |  | CaBR_82 | 68.8 |  | CaBR_82 | 85 |
| CaBR_83 | 1.7 |  | CaBR_83 | 52 |  | CaBR_83 | 52 |
| CaBR_84 | 14.4 |  | CaBR_84 | 85.5 |  | CaBR_84 | 81 |
| CaBR_85 | 0.6 |  | CaBR_85 | 45 |  | CaBR_85 | 51.3 |
| CaBR_86 | 25.6 |  | CaBR_86 | 20 |  | CaBR_86 | 8.5 |
| CaBR_87 | 52.9 |  | CaBR_87 | 55 |  | CaBR_87 | 47 |
| CaBR_89 | 79 |  | CaBR_89 | 53 |  | CaBR_89 | 53 |
| CaBR_90 | 7.8 |  | CaBR_90 | 48.5 |  | CaBR_90 | 48.5 |
| CaBR_91 | 20.5 |  | CaBR_91 | 100 |  | CaBR_91 | 100 |
| CaBR_93 | 25 |  | CaBR_93 | 57 |  | CaBR_93 | 46.3 |
| CaBR_95 | 0.5 |  | CaBR_95 | 71 |  | CaBR_95 | 71 |
| CaBR_96 | 18 |  | CaBR_96 | 89 |  | CaBR_96 | 98.9 |
| CaBR_97 | 13.5 |  | CaBR_97 | 56 |  | CaBR_97 | 56 |
| CaBR_98 | 3.3 |  | CaBR_98 | 2.8 |  | CaBR_98 | 4.4 |
| CaBR_99 | 3.5 |  | CaBR_99 | 22.8 |  | CaBR_99 | 61.4 |
| CaBR_100 | 1.5 |  | CaBR_100 | 70.5 |  | CaBR_100 | 60.5 |
| CaBR_101 | 12 |  | CaBR_101 | 44.5 |  | CaBR_101 | 44.5 |
| CaBR_102 | 9.5 |  | CaBR_102 | 62.5 |  | CaBR_102 | 62.5 |
| CaBR_103 | 10 |  | CaBR_103 | 56.9 |  | CaBR_103 | 56.9 |
| CaBR_104 | 13 |  | CaBR_104 | 21 |  | CaBR_104 | 92.5 |
| CaBR_106 | 27.5 |  | CaBR_106 | 26 |  | CaBR_106 | 26 |
| CaBR_107 | 1.5 |  | CaBR_107 | 95 |  | CaBR_107 | 95 |
| CaBR_108 | 23.5 |  | CaBR_108 | 34 |  | CaBR_108 | 34 |
| CaBR_109 | 53.5 |  | CaBR_109 | 17.5 |  | CaBR_109 | 17.5 |
| CaBR_110 | 15 |  | CaBR_110 | 0 |  | CaBR_110 | 0 |
| CaBR_111 | 60 |  | CaBR_111 | 34.4 |  | CaBR_111 | 24.4 |
| CaBR_112 | 80 |  | CaBR_112 | 39.4 |  | CaBR_112 | 34.4 |
| CaBR_113 | 60 |  | CaBR_113 | 0 |  | CaBR_113 | 0 |
| CaBR_114 | 60.5 |  | CaBR_114 | 28 |  | CaBR_114 | 28 |
| CaBR_115 | 40 |  | CaBR_115 | 35.5 |  | CaBR_115 | 28 |
| CaBR_116 | 72 |  | CaBR_116 | 56 |  | CaBR_116 | 61.5 |
| CaBR_118 | 80 |  | CaBR_118 | 60 |  | CaBR_118 | 42 |
| CaBR_119 | 46 |  | CaBR_119 | 34.5 |  | CaBR_119 | 39 |
| CaBR_120 | 76.3 |  | CaBR_120 | 50 |  | CaBR_120 | 10 |
| CaBR_121 | 49 |  | CaBR_121 | 33.9 |  | CaBR_121 | 32.2 |
| CaBR_123 | 1.5 |  | CaBR_123 | 44.4 |  | CaBR_123 | 63.3 |
|  |  |  | CaBR_125 | 19.5 |  | CaBR_125 | 19.5 |
|  |  |  | CaBR_126 | 53.1 |  | CaBR_126 | 68.8 |
|  |  |  | CaBR_128 | 48.3 |  | CaBR_128 | 21.7 |
| CaBR_129 | 0 |  | CaBR_129 | 25 |  | CaBR_129 | 31.7 |
| CaBR_131 | 0.6 |  | CaBR_131 | 57 |  | CaBR_131 | 80 |
| CaBR_132 | 5.6 |  | CaBR_132 | 51.5 |  | CaBR_132 | 74.5 |
| CaBR_133 | 15 |  | CaBR_133 | 53.9 |  | CaBR_133 | 57.8 |
| CaBR_135 | 3 |  | CaBR_135 | 64.5 |  | CaBR_135 | 64.5 |
| CaBR_137 | 9 |  | CaBR_137 | 43 |  | CaBR_137 | 82 |
| CaBR_138 | 17 |  | CaBR_138 | 50.5 |  | CaBR_138 | 73.5 |
| CaBR_141 | 33.3 |  | CaBR_141 | 41 |  | CaBR_141 | 73 |
| CaBR_143 | 14.5 |  | CaBR_143 | 89 |  | CaBR_143 | 96 |
| CaBR_146 | 4 |  | CaBR_146 | 78 |  | CaBR_146 | 76 |
| CaBR_147 | 17.5 |  | CaBR_147 | 87.5 |  | CaBR_147 | 83.5 |
| CaBR_148 | 14.5 |  | CaBR_148 | 67.5 |  | CaBR_148 | 35 |
| CaBR_149 | 21 |  | CaBR_149 | 90.5 |  | CaBR_149 | 41.5 |
| CaBR_150 | 5.5 |  | CaBR_150 | 58.9 |  | CaBR_150 | 9.5 |
| CaBR_151 | 29 |  | CaBR_151 | 80.6 |  | CaBR_151 | 89.5 |
| CaBR_152 | 9 |  | CaBR_152 | 68.9 |  | CaBR_152 | 33 |
| CaBR_153 | 45.6 |  | CaBR_153 | 81.5 |  | CaBR_153 | 59.5 |
| CaBR_156 | 20 |  | CaBR_156 | 66 |  | CaBR_156 | 45 |
| CaBR_158 | 22 |  | CaBR_158 | 45.5 |  | CaBR_158 | 5 |
| CaBR_159 | 8.5 |  | CaBR_159 | 76.3 |  | CaBR_159 | 30.6 |
| CaBR_160 | 2 |  | CaBR_160 | 74 |  | CaBR_160 | 76.3 |
| CaBR_162 | 19 |  | CaBR_162 | 22.2 |  | CaBR_162 | 22.2 |
| CaBR_163 | 12 |  | CaBR_163 | 61 |  | CaBR_163 | 47.5 |
| CaBR_164 | 11.7 |  | CaBR_164 | 52.5 |  | CaBR_164 | 59 |
| CaBR_165 | 10.6 |  | CaBR_165 | 80.6 |  | CaBR_165 | 67.5 |
| CaBR_168 | 15 |  | CaBR_168 | 57.9 |  | CaBR_168 | 57.9 |
| CaBR_170 | 14 |  | CaBR_170 | 66.9 |  | CaBR_170 | 83.1 |
| CaBR_172 | 3.3 |  | CaBR_172 | 53.1 |  | CaBR_172 | 66.5 |
| CaBR_173 | 43 |  | CaBR_173 | 0 |  | CaBR_173 | 0 |
| CaBR_174 | 0.5 |  | CaBR_174 | 57.2 |  | CaBR_174 | 57.2 |
| CaBR_176 | 1.5 |  | CaBR_176 | 60 |  | CaBR_176 | 55 |
| CaBR_177 | 31.3 |  | CaBR_177 | 93.8 |  | CaBR_177 | 93.8 |
| CaBR_180 | 11.7 |  | CaBR_180 | 0.7 |  | CaBR_180 | 0.7 |
| CaBR_181 | 0.5 |  | CaBR_181 | 28.1 |  | CaBR_181 | 35 |
| CaBR_182 | 2 |  | CaBR_182 | 55.6 |  | CaBR_182 | 86.7 |
| CaBR_184 | 9 |  | CaBR_184 | 51.5 |  | CaBR_184 | 51.5 |
| CaBR_185 | 1 |  | CaBR_185 | 82 |  | CaBR_185 | 82 |
| CaBR_186 | 18 |  | CaBR_186 | 72 |  | CaBR_186 | 72 |
| CaBR_188 | 9.5 |  | CaBR_188 | 91 |  | CaBR_188 | 87.5 |
| CaBR_189 | 32 |  | CaBR_189 | 81 |  | CaBR_189 | 79 |
| CaBR_190 | 8.5 |  | CaBR_190 | 12.5 |  | CaBR_190 | 5 |
| CaBR_192 | 15.5 |  | CaBR_192 | 26.7 |  | CaBR_192 | 39.2 |
| CaBR_193 | 25.5 |  | CaBR_193 | 31.4 |  | CaBR_193 | 28.6 |
| CaBR_194 | 6.5 |  | CaBR_194 | 22.5 |  | CaBR_194 | 34 |
