# Supplementary material for: A GBS-based genetic linkage map and quantitative trait loci (QTL) associated with resistance to Xanthomonas campestris pv. campestris race 1 identified in Brassica oleracea
Source: Front Plant Sci. 2023 Jun 13;14:1205681. doi: 10.3389/fpls.2023.1205681 (PMC10293835; doi:10.3389/fpls.2023.1205681)
Supplement: Supplementary file 4 [file Table_3.docx]

**Table S3**. Summary of whole-genome resequencing data for *B. olerecea* lines

|  | **BR155** | **SC31** |
| --- | --- | --- |
| Raw read | 68,892,465 | 67,851,038 |
| Raw bases | 10,402,762,215 | 10,245,506,738 |
| Coverage of *B. olerecea* genome | 24.22 X | 24.29 X |
| Mapped reads | 86,106,852 | 90,141,562 |
| Mapped percentage (%) | 70.74 | 74.67 |
| Mapped region (%) | 72.44 | 78.39 |
