# Supplementary material for: A GBS-based genetic linkage map and quantitative trait loci (QTL) associated with resistance to Xanthomonas campestris pv. campestris race 1 identified in Brassica oleracea
Source: Front Plant Sci. 2023 Jun 13;14:1205681. doi: 10.3389/fpls.2023.1205681 (PMC10293835; doi:10.3389/fpls.2023.1205681)
Supplement: Supplementary file 5 [file Table_4.docx]

**Table S4.** A list of genes with description located in the major QTL interval.

| ***B. oleracea*_ID** | **Annotation (Functional description)** | **Arabidopsis_ID** |
| --- | --- | --- |
| Bo6g095580 | Chorismate mutase 3 | AT1G69370 |
| Bo6g095810 | Defensin-like (DEFL) family protein | AT1G68905 |
| Bo6g095900 | Phosphoenolpyruvate carboxylase 4 | AT1G68750 |
| Bo6g096080 | 3-ketoacyl-CoA synthase 6 | AT1G68530 |
| Bo6g097240 | B-box zinc finger family protein | AT1G68190 |
| Bo6g097250 | Ubiquitin-like superfamily protein | AT1G68185 |
| Bo6g097340 | 2-oxoglutarate (2OG) and Fe(II)-dependent oxygenase superfamily protein | AT1G68080 |
| Bo6g097360 | UDP-Glycosyltransferase / trehalose-phosphatase family protein | AT1G68020 |
| Bo6g098450 | Phototropic-responsive NPH3 family protein | AT1G67900 |
| Bo6g098480 | ß-1,4-N-acetylglucosaminyltransferase family protein | AT1G67880 |
| Bo6g098570 | Chloroplast sensor kinase | AT1G67840 |
| Bo6g098910 | Recovery protein 3 | AT1G67500 |
| Bo6g098920 | Glucosidase 1 | AT1G67490 |
| Bo6g098930 | Expressed protein | AT2G14247 |
| Bo6g099080 | ARM repeat superfamily protein | AT1G64960 |
| Bo6g099190 | 4-coumarate:CoA ligase 3 | AT1G65060 |
| Bo6g099240 | Cystathionine beta-synthase (CBS) family protein | AT1G65320 |
| Bo6g099300 | HXXXD-type acyl-transferase family protein | AT1G65450 |
| Bo6g099310 | chromatin assembly factor-1 (FASCIATA1) (FAS1) | AT1G65470 |
| Bo6g099330 | LETM1-like protein | AT1G65540 |
| Bo6g099340 | Pectin lyase-like superfamily protein | AT1G65570 |
| Bo6g099350 | Endonuclease/exonuclease/phosphatase family protein | AT1G65580 |
| Bo6g099400 | ß-hexosaminidase 3 | AT1G65590 |
| Bo6g099420 | Peptidase C12%2C ubiquitin carboxyl-terminal hydrolase 1 | AT1G65650 |
| Bo6g099480 | YELLOW STRIPE like 7 | AT1G65730 |
| Bo6g099490 | P-loop containing nucleoside triphosphate hydrolases superfamily protein | AT1G65780 |
| Bo6g099600 | Protein kinase superfamily protein | AT1G65950 |
| Bo6g099640 | Nucleic acid-binding%2C OB-fold-like protein | AT5G38890 |
| Bo6g099660 | Cysteine/Histidine-rich C1 domain family protein | AT1G34480 |
| Bo6g099750 | Receptor-like transmembrane kinase I (TMK1) | AT1G66150 |
| Bo6g099790 | Subtilase family protein | AT1G66220 |
| Bo6g099800 | MYB domain protein 20 | AT1G66230 |
| Bo6g099830 | RNA-binding (RRM/RBD/RNP motifs) family protein | AT1G66260 |
| Bo6g099850 | ETHYLENE INSENSITIVE 1 | AT1G66340 |
| Bo6g100950 | Calmodulin like 23 | AT1G66400 |
| Bo6g100960 | Calmodulin 4 | AT1G66410 |
| Bo6g100970 | Protein kinase superfamily protein | AT1G66460 |
| Bo6g100980 | pfkB-like carbohydrate kinase family protein | AT1G66430 |
| Bo6g101010 | Plastid movement impaired 2 | AT1G66480 |
| Bo6g101030 | AAR2 protein family | AT1G66510 |
| Bo6g101040 | Formyltransferase | AT1G66520 |
| Bo6g101060 | Protein with RING/U-box and TRAF-like domains | AT1G66630 |
| Bo6g101120 | S-adenosyl-L-methionine-dependent methyltransferases superfamily protein | AT1G66680 |
| Bo6g101210 | DNA LIGASE 6 | AT1G66730 |
| Bo6g101230 | CDK-activating kinase 4 | AT1G66750 |
| Bo6g101240 | MATE efflux family protein | AT1G64820 |
| Bo6g101310 | Leucine-rich repeat protein kinase family protein | AT1G66830 |
| Bo6g101340 | Disease resistance protein (TIR-NBS-LRR class) family | AT5G46450 |
| Bo6g103520 | zinc finger protein 6 | AT1G67030 |
| Bo6g103590 | HEAT repeat-containing protein | AT1G67140 |
| Bo6g103610 | F-box/RNI-like superfamily protein | AT1G67190 |
| Bo6g103690 | Dioxygenase superfamily protein | AT1G67280 |
| Bo6g103710 | Major facilitator superfamily protein | AT1G67300 |
| Bo6g103870 | Leucine-rich repeat protein kinase family protein | AT1G67510 |
| Bo6g103930 | Cysteine/Histidine-rich C1 domain family protein | AT5G01480 |
| Bo6g103970 | Protein kinase superfamily protein | AT1G67580 |
| Bo6g104000 | Lojap-related protein | AT1G67620 |
| Bo6g104010 | F-box family protein | AT1G67623 |
| Bo6g104030 | Restriction endonuclease type II-like superfamily protein | AT1G67660 |
| Bo6g105130 | Brassinosteroid-signaling kinase 11 | AT1G50990 |
| Bo6g105140 | FTSH protease 1 | AT1G50250 |
| Bo6g105150 | Leucine-rich repeat protein kinase family protein | AT1G67720 |
| Bo6g105230 | Terminal EAR1-like 2 | AT1G67770 |
| Bo6g106270 | Copine (Calcium-dependent phospholipid-binding protein) family | AT1G67800 |
| Bo6g106430 | Protein POLLEN DEFECTIVE IN GUIDANCE 1 | AT1G67960 |
| Bo6g106440 | RNA-binding (RRM/RBD/RNP motifs) family protein | AT1G67950 |
| Bo6g106700 | Endonuclease 2 | AT1G68290 |
| Bo6g106710 | Adenine nucleotide alpha hydrolases-like superfamily protein | AT1G68300 |
| Bo6g106780 | Chaperone DnaJ-domain superfamily protein | AT1G68370 |
| Bo6g106790 | Core-2/I-branching ß-1,6-N-acetylglucosaminyltransferase family protein | AT1G68380 |
| Bo6g106900 | Exostosin family protein | AT1G68470 |
| Bo6g106960 | LOB domain-containing protein 42 | AT1G68510 |
| Bo6g106980 | NAD(P)-binding Rossmann-fold superfamily protein | AT1G68540 |
| Bo6g107110 | PLANT CADMIUM RESISTANCE 11 | AT1G68610 |
| Bo6g107140 | bZIP transcription factor family protein | AT1G68640 |
| Bo6g107190 | Arabinogalactan protein 19 | AT1G68725 |
| Bo6g107220 | RNI-like superfamily protein | AT1G68780 |
| Bo6g108280 | Transmembrane Fragile-X-F-associated protein | AT1G68820 |
| Bo6g108290 | STT7 homolog STN7 | AT1G68830 |
| Bo6g108490 | Armadillo/beta-catenin-like repeat family protein | AT1G68940 |
| Bo6g108540 | Prolyl oligopeptidase family protein | AT1G69020 |
| Bo6g108580 | Chaperone DnaJ-domain superfamily protein | AT1G69060 |
| Bo6g108590 | Adenine nucleotide alpha hydrolases-like superfamily protein | AT1G69080 |
| Bo6g108630 | Plant-specific transcription factor YABBY family protein | AT1G69180 |
| Bo6g108640 | Fructokinase-like 2 | AT1G69200 |
| Bo6g108770 | RING/U-box superfamily protein | AT1G69330 |
| Bo6g108780 | Tetratricopeptide repeat (TPR)-like superfamily protein | AT1G69350 |
| Bo6g108830 | Eukaryotic elongation factor 5A-3 | AT1G69410 |
| Bo6g108870 | Early-responsive to dehydration stress protein (ERD4) | AT1G69450 |
| Bo6g108890 | Transmembrane emp24 domain-containing protein p24delta10 | AT1G69460 |
| Bo6g108910 | EXS (ERD1/XPR1/SYG1) family protein | AT1G69480 |
| Bo6g108940 | Cytochrome P450, family 704 | AT1G69500 |
| Bo6g108950 | cAMP-regulated phosphoprotein 19-related protein | AT1G69510 |
| Bo6g108990 | S-adenosyl-L-methionine-dependent methyltransferases superfamily protein | AT1G69520 |
| Bo6g109020 | S-adenosyl-L-methionine-dependent methyltransferases superfamily protein | AT1G69523 |
| Bo6g109050 | AGAMOUS-like 94 | AT1G69540 |
