# Supplementary material for: A GBS-based genetic linkage map and quantitative trait loci (QTL) associated with resistance to Xanthomonas campestris pv. campestris race 1 identified in Brassica oleracea
Source: Front Plant Sci. 2023 Jun 13;14:1205681. doi: 10.3389/fpls.2023.1205681 (PMC10293835; doi:10.3389/fpls.2023.1205681)
Supplement: Supplementary file 6 [file Table_5.docx]

**Table S5**. The list of primer sequences used for qPCR

| ***B. oleracea* ID** | **Left-Primer** | **Right-Primer** | **Product size (bp)** |
| --- | --- | --- | --- |
| Bo6g095580 | GGGGATCTTTGGTCGAGTTT | GGCGCAATGATGTAGAACCT | 158 |
| Bo6g098480 | TCACGGCCTCAAGATCTCAT | ACTTCGGTGCTGAAGAGGAC | 183 |
| Bo6g099850 | CCGTCGCCTACTTCTCAATC | ATGGGTGGTGAAGGTCCATA | 152 |
| Bo6g101010 | GTCAAACGAGCGTGAGGTTT | AAGTTTCCGGTGGTTGTCAC | 152 |
| Bo6g101210 | CTCCAAGTCCCGTCACAACT | TCATCGAATCGCAAAATCTG | 184 |
| Bo6g101310 | TTTGACCTCGACCAGCTTCT | GATGCTTGATTTTCGCCATT | 184 |
| Bo6g106440 | TTGACGAGCGGTATCAAGTG | CTTCCCTCTCCTCCATCCTC | 184 |
| Bo6g108870 | ACGCAACAAAGTCGCTAGGT | GATCACCGAAGACGTTGACA | 200 |
